# Biomarkers in Advanced Colorectal Cancer: Challenges in Translating Clinical Research into Practice

**DOI:** 10.3390/cancers3021844

**Published:** 2011-04-01

**Authors:** Charlotte Lemech, Hendrik-Tobias Arkenau

**Affiliations:** Sarah Cannon Research Institute, 93 Harley Street, W1G 6AD, London, UK; E-Mail: charlotte.lemech@sarahcannonresearch.co.uk (C.L.)

**Keywords:** prognostic biomarker, predictive biomarker, colorectal cancer, kras mutation

## Abstract

The growing number of therapeutic agents and known molecular targets in oncology makes the study and clinical use of biomarkers imperative for improving response and survival, reducing toxicity and ensuring economic sustainability. Colorectal cancer, among others, is at the forefront of development of predictive and prognostic biomarkers; however, the difficulty lies in translating potential biomarkers garnered from retrospective analyses in small numbers of patients to generalizable and affordable biomarkers used worldwide. This review outlines the progress made in prognostic and predictive biomarkers in advanced colorectal cancer (ACRC) from the early use of carcinoembryonic antigen (CEA) to the KRAS mutation and beyond. Future challenges are to incorporate standardized and validated methods preferentially during early phases of drug development linked with sophisticated biostatistical support. New trial designs focusing on biomarkers will be essential not only for better understanding of mechanisms of action, but also to make confident ‘go or no-go’ decisions.

## 1. Introduction

Colorectal cancer is the third most common cancer among men and women and the second most common cause of cancer-related death among patients in the United Kingdom [1]. The prognosis of advanced colorectal cancer (ACRC) has improved significantly over the last decade due to an increased efficacy and availability of chemotherapy and biological agents.

Intravenous or oral fluoropyrimidine chemotherapies form the backbone of treatment in combination with oxaliplatin or irinotecan [2]. In addition, biological agents are commonly added to these regimens to increase response rate and survival and to achieve down-staging for surgical resection and potential cure.

The currently approved and widely used biological agents are the monoclonal antibodies bevacizumab, targeting the vasculature endothelial growth factor (VEGF) and cetuximab and panitumumab, targeting the epidermal growth factor receptor (EGFR).These combinations achieve response rates (RR) of approximately 50% with a median time of progression free survival (PFS) of 10–12 months in patients with ACRC.

In the era of personalized medicine, predictive and prognostic biomarkers are increasingly important in tailoring treatment decisions for individual patients. Despite better understanding of tumor biology and improved diagnostic technology, challenges remain to predict response and tolerability to new treatments and more importantly, survival. Further research is essential not only for improving clinical outcome for patients with ACRC, but also for reducing side effects and maintaining economic sustainability.

## 2. Early Prognostic and Predictive Biomarkers

Biomarkers are often used and objectively measured to evaluate pathological processes or pharmacological responses to a therapeutic intervention [3], and can be any kind of molecule, substance, or genetic marker which is traceable. Predictive biomarkers provide information on response to a treatment, whereas prognostic biomarkers give information about outcome independent of the treatment effect.

The carcinoembryonic antigen (CEA) is one of the earliest studied biomarkers in colorectal cancer and has a role in surveillance after treatment for early stage disease [4]. CEA is also used in monitoring patients with advanced disease receiving palliative chemotherapy, as recommended in the updated 2006 ASCO guidelines [5], although this is not advocated by all [6]. Postoperative CEA has also been shown to be a prognostic factor after resection of colorectal liver metastases [7]. CEA however lacks sensitivity and specificity and thus in many settings can be a poor prognostic and predictive factor. Even in ACRC, up to 30% of patients may have a normal CEA [8]. In fact CEA is also elevated in a number of benign conditions as well as in heavy smokers, with an elevated reading in up to 13.6% of heavy smokers compared to 1.8% of non-smokers [9]. Thus, CEA should be interpreted with caution but can be useful on a case by case basis.

The microsatellite instability (MSI) status has also been studied as both a prognostic and predictive marker in colorectal cancer. MSI refers to a clonal change in the number of repeated DNA nucleotide units in microsatellites [10] and occurs in tumors with deficient mismatch repair due to inactivation of one of the four mismatch repair (MMR) genes; MSH2, MLH1, MSH6 and PMS2. Lynch syndrome (3–5% of all CRCs) is characterized by germline mutations of the mismatch repair genes while a further 10–15% of colorectal cancers will have sporadic mutations [11]. This is found in 22% of stage 2 CRCs, 12% of stage 3 CRCs [12], but only 3.5% of ACRC [13]. MSI positive tumors are associated with a more favorable prognosis [14] in all stages of disease; however, its predictive utility is predominantly in the adjuvant setting [15-17].

## 3. Prognostic and Predictive Biomarkers in the Setting of EGFR Directed Therapies

The EGFR pathway plays an important role in tumor growth through regulation of proliferation, angiogenesis, invasion and metastasis. It is mediated by downstream pathways including the RAS-RAF-mitogen-activated protein kinase (MAPK) and phosphatidylinositol 3-kinase (PI3K)-AKT-mTOR pathways [18,19].

Treatment with EGFR monoclonal antibodies was initially based on EGFR over-expression, assessed by immunohistochemistry (IHC) on formalin-fixed paraffin-embedded tumour specimens [20].

Cetuximab, a chimeric mouse-human monoclonal IgG1 antibody to EGFR, and panitumumab, a fully human monoclonal IgG2 antibody to EGFR, have shown improved response rate (RR), progression free survival (PFS) and overall survival (OS) both when used as monotherapy for refractory ACRC or in combination with chemotherapy (Table 1).

The BOND study [20] was the first to report an improved RR, PFS and OS in patients with prior progression on irinotecan based chemotherapy randomized to cetuximab monotherapy or cetuximab plus irinotecan. In the monotherapy arm, the RR was 10.8% with stable disease (SD) in an additional 21.6% of patients, whereas in the combination arm the RR was 22.9% with 32.6% SD. Interestingly, it was found that patients with ‘skin rash’ had non-significant higher RR. This pivotal trial guided the initial FDA approval in 2004 for cetuximab in EGFR expressing ACRC in patients who were refractory to or intolerant of irinotecan-based chemotherapy [21].

The National Cancer Institute of Canada Clinical Trials Group CO.17 Trial (NCIC CO-17) demonstrated both improved OS and preservation of quality of life in patients with refractory ACRC treated with cetuximab monotherapy compared to best supportive care. The RR was 8.0% and disease stabilization was seen in an additional 31.4%. Again, patients were enrolled based on EGFR IHC over-expression, which did not correlate with response, but a correlation was found between the severity of ‘skin rash’ and OS [22].

Similarly, panitumumab monotherapy led to a PFS benefit in EGFR IHC positive ACRC patients who progressed after standard chemotherapy. The RR was 10%, and a further 27% achieved SD. Although, there was an association between clinical efficacy and ‘skin rash’ severity, this study also identified that ‘skin rash’ was not always correlated with response and could occur in patients who did not benefit from treatment [23].

With the observed association between ‘skin rash’ and response in early studies and its potential use as a predictive biomarker, a prospective study assessed the role of high dose cetuximab and expression of ‘skin rash’ in patients who had prior progression on irinotecan. After three weeks of treatment patients with no or mild skin rash received increased doses of cetuximab to explore whether higher dosing could result in skin rash and subsequently higher RR [24]. Interestingly, higher dosing did result in increased RR, however this did not translate into a PFS or OS benefit, supporting previous findings that ‘skin rash’ might not be an ideal biomarker for response. Furthermore the ‘Skin Toxicity Evaluation Protocol with Panitumumab’ (STEPP) trial investigated the role of ‘pre-emptive’ (protective skin moisturizers, sunscreen, topical steroid, and doxycycline) versus ‘reactive’ management of skin rash. The incidence of protocol-specified ≥ grade 2 skin rash was 29% and 62% for the pre-emptive and reactive groups, respectively. However in terms of clinical outcome there was no difference between both groups indicating the limitations of ‘skin rash’ as a predictive biomarker for response [25].

In the search for a biomarker to better predict response to cetuximab several studies looked at positive and negative EGFR IHC expression, and in addition at EGFR gene copy number and EGFR mutational status. In this context two studies confirmed that even EGFR IHC negative ACRCs had clinical response to cetuximab [26,27]. Moreover EGFR mutation status and EGFR gene copy number did not show an association with response and EGFR expression [28].

The importance of Kirsten rat sarcoma-2 virus oncogene (KRAS) mutation and an increased understanding of the complex EGFR downstream signaling cascade were the first steps in identifying predictive biomarkers for EGFR directed therapies in patients with ACRC.

Mutations in KRAS can cause ongoing activation of the downstream RAS-RAF-MAPK and PI3K-AKT-mTOR pathways, regardless of whether the upstream EGFR is activated or blocked. Between 35–45% of ACRC have been shown to bear KRAS mutations, most commonly in codons 12 and 13 [29-31].

Initial retrospective cohort studies of KRAS mutations in early stages of colorectal cancer indicated a prognostic significance. The ‘Kirsten ras mutations in patients with colorectal cancer’ (RASCAL) study investigated 2721 tumor samples and their KRAS mutational status. Multivariate analysis suggested that the presence of a mutation increased the risk of recurrence and death—in particular the glycine to valine mutation in codon 12 [32]. The prognostic role of KRAS was further supported in the larger RASCAL II study including 3439 patients with Dukes C tumors [33].

In the context of ACRC the Medical Research Council (MRC) Focus trial investigated the role of KRAS as a prognostic, but also predictive biomarker in patients who underwent fluoropyrimidine based chemotherapy with either oxaliplatin or irinotecan. In this study the presence of KRAS mutation was associated with a shorter OS, however minimal impact on PFS and no effect on the clinical impact of irinotecan or oxaliplatin treatment was seen [34], indicating that KRAS had no predictive role in ACRC patients on standard chemotherapy.

The first association between KRAS mutational status and response to EGFR-antibody therapy was reported by Lievre *et al.* [30]. In this study 43% of all patients had a KRAS mutation and there were no responses to cetuximab in this group. However, patients with wildtype (wt) KRAS, had a response rate of 65%. An increased EGFR gene copy number, although found in only 10%, was also significantly associated with increased RR (Table 2).

The larger NCIC C0-17 study confirmed a KRAS mutation rate of >40% in patients with ACRC and in addition found that KRAS mutational status was a negative predictor of PFS for patients who underwent cetuximab based therapy [35]. In the patient cohort who received best supportive care there was no difference in OS regardless of KRAS mutational status, supporting the notion that KRAS had no prognostic, but only predictive value in this setting.

Another study confirmed that ACRC wtKRAS patients had improved RR and PFS with panitumumab monotherapy as opposed to patients with KRAS mutations [36].

The addition of panitumumab to FOLFOX as first line treatment and to FOLFIRI as second line treatment also showed an improvement in RR and PFS in wtKRAS patients [37,38]. Similarly, cetuximab also demonstrated improved RR and PFS in wtKRAS patients in combination with first-line and second-line chemotherapy (Table 1) [39-41].

In contrast, a recent analysis of the MRC COIN trial demonstrated no added benefit of cetuximab to standard oxaliplatin based first-line chemotherapies in wtKRAS ACRC patients [42]. Further subgroup analyses are underway to identify factors which might have impacted on these results.

In fact, although the identification of KRAS mutational status identifies a significant subgroup of patients who do not respond to EGFR inhibitors, there are a number of other downstream signals which further modulate its effect, as evidenced by up to 30–40% of patients who do not respond to EGFR inhibition despite being wtKRAS.

## 4. Beyond KRAS and Challenges of New Biomarker Development

Activation of KRAS can activate both the MAPKinase signaling pathway and the PIK3-AKT-mTOR pathway [18,19]. Mutations, gene amplification and loss of tumor suppressor genes in both pathways can result in further downstream signaling.

Retrospective analyses of the BRAF gene have indicated that mutations in this gene have a role as a negative prognostic marker in ACRC (Table 2). Di Nicolantonio *et al.* first established that wtBRAF was necessary for response to EGFR inhibitors [43]. Patients with wtKRAS who received cetuximab responded only if they had also wtBRAF, whereas a small number of patients (14% of the tumors) with wtKRAS but mtBRAF had lower RR, PFS and OS. Importantly this study also found that KRAS and BRAF mutations were mutually exclusive.

Results of a recent pooled analysis of the two CRYSTAL and OPUS trials showed improved OS with cetuximab and chemotherapy in wtKRAS and wtBRAF ACRC patients compared to chemotherapy alone (24.8 months *versus* 21.1 months) [44]. Cetuximab increased the median OS from 9.9 to 14.1 months for patients who were wtKRAS but mtBRAF demonstrating that mtBRAF is a prognostic factor. Moreover, patients with wtKRAS and mtBRAF still seem to benefit from cetuximab with chemotherapy and treatment decisions regarding the use of cetuximab should not be made based on the presence solely of BRAF mutational status.

There is preclinical and early clinical evidence that phosphatase and tensin homolog (PTEN) gene loss confers resistance to EGFR inhibitors and allows persistent downstream activation via the AKT-mTOR axis [45-47]. Loss of expression of PTEN occurs by a number of means including promoter methylation [48], microRNA suppression [49] and PTEN mutation. PTEN loss is measured by immunohistochemistry and due to the lack of standardized methodology and validated assays there can be inter-laboratory variation, such that other techniques including fluorescent *in situ* hybridization are currently under investigation [45].

Laurent-Puig *et al.* assessed PTEN, KRAS, BRAF and EGFR status in ACRC patients who received chemotherapy in combination with cetuximab (Table 2) [46]. This analysis confirmed that wtKRAS but mtBRAF tumors were associated with lack of response, shorter PFS and OS. In addition loss of PTEN was demonstrated in 20% of wt KRAS tumors. In this subgroup PTEN loss was associated with shorter OS, however not with reduced RR or PFS. Interestingly PTEN loss could occur in both mtBRAF or mtKRAS tumors.

An analysis by Loupakis *et al.* found PTEN loss in nearly 40% of wtKRAS tumours and PTEN loss was associated with a lack of response to cetuximab and irinotecan whereas patients with wtKRAS and normal PTEN status had improved RR and PFS [47]. Importantly the concordance between primary tumours and metastases was 60% for PTEN compared to 95% for mtKRAS indicating the need for fresh tumour biopsies prior to treatment decisions.

In addition to BRAF mutations and PTEN loss, NRAS mutations have been identified in 3-5% of wtKRAS patients and clinical data confirm lack of response to EGFR antibody therapy [50]. Furthermore PIK3CA mutations have been reported in the range of 6% to 40% [51-53]. An association between PIK3CA mutations and lack of response to EGFR directed therapy has been reported by Sartore-Bianchi *et al.* however these results were not in accordance with other studies [54-57]. Certainly the retrospective nature and low sample size, but also the exon location analysis of the PI3K mutation may have impacted on these results and further clarification is warranted [54].

In addition to gene mutation analysis, gene expression profiling may also identify predictive factors. Khambata-Ford *et al* have demonstrated that high gene expression levels of amphiregulin and epiregulin also correlate with better response to cetuximab [58]. Amphiregulin and epiregulin are ligands of EGFR and high gene expression is thought to reflect greater dependence of tumor growth on the EGF pathway and thus, greater susceptibility to EGFR inhibition. It is thought that elevated expression of epiregulin and/or amphiregulin may stimulate an autocrine loop through EGFR thus promoting tumor growth and survival. Gene expression profiling may thus give additional predictive information, along with mutation status of KRAS, NRAS, BRAF, PIK3CA and PTEN loss.

## 5. Biomarkers for VEGF Directed Therapies

Angiogenesis is an essential component of tumor growth and vascular endothelial growth factors and receptors (VEGF and VEGFR) play a critical role in this process [59,60]. Bevacizumab has shown improved clinical outcome when added to first and second-line chemotherapy, however there is still debate about the magnitude of clinical benefit patients with ACRC can derive [61]. Although bevacizumab is widely regarded as standard treatment, several health care systems have not approved this drug because of the limited cost-benefit ratio. The search for an appropriate easily derivable predictive biomarker to select patients who most likely benefit from this treatment has been disappointing. So far clinical, radiological and molecular methods have been assessed unsuccessfully [62].

Clinically, correlation between hypertension and PFS and OS has been found in several phase 3 trials of bevacizumab, particularly in non small cell lung cancer, breast cancer and metastatic renal cell cancer [63-65]. However, a meta-analysis by Hurwitz et al of six trials in colorectal, breast and renal cell cancer, showed that the development of hypertension predicted improved OS and PFS in only one study [66].

Various imaging modalities have yielded some promising preliminary data, but are yet to be validated in larger studies. Morphological CT imaging, dynamic contrast enhanced (DCE)-MRI and 18F-fluorothymidine (FLT)-PET have all been assessed in small studies in ACRC with liver metastases suggesting a better correlation with PFS and OS compared to the standard Response Evaluation Criteria in Solid Tumors (RECIST) [67-69]. These imaging modalities however have been criticised as not being cost effective nor time efficient in day-to-day practice.

Studies of circulating VEGF, circulating endothelial cells (CEC) and peripheral blood neutrophil count have had mixed results. Although attempts have been made to assess circulating VEGF levels in both retrospective and prospective trials, the utility of a predictive biomarker have been limited by a number of factors. VEGF is a dynamic marker that changes with treatment and many studies were flawed by the selection of sampling time points before and during treatment [70]. Secondly, both free and bound VEGF levels may need evaluation as more than 98% of circulating VEGF may be bound to antibody after commencing bevacizumab [71]. Other markers such as circulating endothelial cells (CECs), thought to reflect active angiogenesis, have been studied in ACRC in small studies and appeared to have predictive value in patients receiving bevacizumab first-line [72]. There is evidence that CECs also have prognostic significance and thus, predictive results are difficult to interpret [73]. Peripheral blood neutrophil count and assessment of other inflammatory cell markers have also been studied in breast, lung and renal cell carcinoma and are thought to reflect VEGF-independent proangiogenic pathways but require validation in larger, prospective ACRC trials [74-76].

Interestingly VEGF gene polymorphisms (VEGF-2578AA and VEGF-1154A) have recently shown some promising results in predicting OS with bevacizumab based treatments in various tumors including ACRC. A study of 285 ACRC patients who received either FOLFIRI or CAPIRI plus bevacizumab demonstrated a significant correlation between VEGF genotype and survival [77].

## 6. Conclusions

In the era of molecular targeted therapies, treatment decisions rely increasingly on molecular profiling where defined molecular signatures predict treatment outcome. As witnessed with EGFR-antibody treatments in patients with ACRC we have observed a shift from ‘one size fits all’ to selected patient groups who derive most benefit from these treatments. Beyond KRAS, other biomarkers including BRAF, PTEN, and PIK3CA will be part of screening panels to identify the true “quadruple negative tumors” responding to EGFR-therapies [78]. Other components of the EGFR pathway including AKT, mTOR, MEK and ERK will yield further knowledge in particular on crosstalk between pathways, mechanisms of resistance and new targets for drug development. In this context the lessons learnt from historical studies should be incorporated to improve patient outcome.

Despite the recent progress, interpretation of current data is still limited by retrospective analyses, single center experience, small sample size and lack of standardization of diagnostic tools. The incorporation of biomarker research into early clinical trials is mandatory to improve development of new often high cost drugs. In addition to the complex process of biomarker development there is also a demand for improved bio-statistical input requiring funding to be focused on multi-institutional collaborative research groups with expertise and capacity to undertake such research.

Consideration of the appropriate sample size for biomarker analysis should also be incorporated into trial design in particular in randomized phase-II studies. Tumor specimen should be pre-specified in clinical trials to reduce the dis-concordance between primary tumor and metastasis as demonstrated by PTEN status in patients with ACRC. Moreover trials may need to incorporate specimens, not only of the primary tumor and metastasis, but also of biopsies of lesions on progression. Finally, as evidenced by the assessment of circulating biomarkers for bevacizumab, standardized timing of blood samples will be crucial depending on the mechanism of action of the drug.

Biomarker development in patients with ACRC came a long way in recent years. Future challenges are to incorporate standardized and validated methods preferentially during early phases of drug development linked with sophisticated bio-statistical support. New trial designs focusing on biomarkers will be essential not only for better understanding of the mechanisms of action, but also to make confident ‘go or no-go decisions’.

## Figures and Tables

**Table 1.** Randomized clinical trials of cetuximab and panitumumab in patients with metastatic colorectal cancer.

**Trial**	**Treatment (line)**	**Patient number (with KRAS results)**	**Initial Biomarker (retrospective marker)**	**Overall Response rate (mt v wt KRAS with EGFRI)**	**Overall PFS and OS (Hazard ratio)**	**PFS wt KRAS: control *vs.* EGFRI (Hazard ratio)**	**PFS mt KRAS: control *vs.* EGFRI (Hazard ratio)**	**OS wt KRAS: control *vs.* EGFRI (Hazard ratio)**	**OS mt KRAS: control *vs.* EGFRI (Hazard ratio)**	**Rash**
BOND [20]	Cetuximab *vs.* irinotecan/cetuximab (third)	329	EGFR IHC+	10.8% *vs.* 22.9% (1.2% *vs.* 12.8%)	PFS 1.5 *vs.* 4.1 m (0.69) p < 0.001 OS 6.9 *vs.* 8.6 m (0.91) p = 0.48					Increase response rate ass'd with skin rash
NCIC CO-17 phase 3 [22,35]	BSC *vs.* cetuximab (third)	572 (394)	EGFR IHC+(kras)	0 *vs.* 8%	OS 4.6 *vs.* 6.1m (0.77) p = 0.005	1.9 *vs.* 3.7 m (0.40) *p* < 0.001	1.8 *vs.* 1.8 m (0.99) sp = 0.96	4.8 *vs.* 9.5 m (0.55) p < 0.001	4.6 *vs.* 4.5 m (0.98) p = 0.89	Rash ass'd with improved survival
200204 8 phase 3 trial [23,36]	BSC *vs.* panitumumab (third)	463 (427)	EGFR IHC+(kras)	0 *vs.* 10% (0 *vs.* 17%)	PFS 7.3 *vs.* 8 wks (0.54) p < 0.001	7.3 *vs.* 12.3 wks (0.45) p < 0.001	7.3 *vs.* 7.4 wks (0.99)	7.6 *vs.* 8.1 m (0.99)	4.4 *vs.* 4.9 m (1.02)	Worse grade rash ass'd with better PFS and OS. Rash occurre din pts without benefit
EPIC phase 3 [39]	Irinotecan +/− cetuximab (second)	1298 (300)	EGFR IHC+ (kras)	4.2% *vs.* 16.4%	PFS 2.6 *vs.* 4.0 m OS 10 *vs.* 10.7 m (0.98) p = 0.71	2.8 *vs.* 4.0m (0.77) p = 0.095	2.7 *vs.* 2.6m (1.00) p = 0.98	11.6 *vs.* 10.9m (1.29) p = 0.18	10.7 *vs.* 8.4 (1.28) p = 0.29	
181 phase 3 [38]	FOLFIRI +/− panitumumab (second)	1186 (1083)	No EGFR IHC criteria Kras prospective	10% *vs.* 35% WT (13% *vs.* 35%)		3.9 *vs.* 5.9 m (0.73) p = 0.004	4.9 *vs.* 5.0 m (0.85) p = 0.14	12.5 *vs.* 14.5 m (0.85) p = 0.12	11.1 *vs.* 11.8m (0.94) p = 0.55	
PRIME phase 3 [37]	FOLFOX4 +/− panitumumab (first)	1183 (1096)	No EGFR IHC criteria Kras prospective	48% *vs.* 55% WT (40% *vs.* 55%)		8.0 *vs.* 9.6 m (0.8) p = 0.02	8.8 *vs.* 7.3 m (1.29) p = 0.02	19.7 *vs.* 23.9 m (0.83) p = 0.07	19.3 *vs.* 15.5 m (1.24) p = 0.07	
CRYS TAL phase 3 [40]	FOLFIRI +/− cetuximab (first)	1198 (1063)	EGFR IHC+(kras)	38.7% *vs.* 46.9% (31.3% *vs.* 59.3%)	PFS 8.0 *vs.* 8.9 m (0.85) p = 0.048 OS 18.6 *vs.* 19.9 m	8.4 *vs.* 9.9 m (0.70) p = 0.001	7.7 *vs.* 7.4 m (1.17) p = 0.276	20 *vs.* 23.5 m (0.80) p = 0.009	16.7 *vs.* 16.2 m (1.04) p = 0.755	
OPUS phase 3 [41]	FOLFOX4 +/− cetuximab (first)	337 (315)	EGFR IHC+(kras)	36% *vs.* 46% p = 0.64 (32.8% *vs.* 57.3%)	PFS 7.2 *vs.* 7.2 m	7.2 *vs.* 8.3 m (0.567) p = 0.006	8.5 *vs.* 5.5m (1.720) p = 0.015	18.5 *vs.* 22.8 m (0.855) p = 0.385	17.5 *vs.* 13.4 m (1.290) p = 0.200	
MRC COIN [42]	FOLFOX/CAPEOX +/− cetuximab (first)	(1305)	Kras prospective	50% *vs.* 59% WT (40% *vs.* 59%)		8.6 *vs.* 8.6 m (0.959) p = 0.60	6.9 *vs.* 6.5 (1.065) p = 0.46	17.9 *vs.* 17.0 (1.04) p = 0.68	14.8 *vs.* 13.6 m (0.98) p = 0.8	

EGFRI = epidermal growth factor receptor inhibitor; mt = mutant; wt = wild-type; IHC = immunohistochemistry; RR = response rate; PFS = progression free survival; OS = overall survival.

**Table 2.** Trials assessing downstream mutations in the EGFR pathway and resistance to the EGFR monoclonal antibodies.

**Trial**	**Patient Number**	**Wt KRAS (%)** **Responders (%)**	**Mt KRAS (%)**	**BRAF mutation Association**	**PTEN loss of expression Association**	**PIK3CA mutation Association**	**Other**
Lievre *et al.*	30	17 (63%) Res 11/17 (65%)	13 (43%)	0	na	2 (7%) [in KRAS mt pts]	EGFR copy number (3 pts-10%) correlates with response
Di Nicolantonio *et al.*	113	79 (70%) Res 22/79 (28%)	34 (30%)	11/79 (10%) Shorter PFS (p = 0.011) and OS (p < 0.0001)	na	na	Sorafenib restored sensitivity to EGFR mabs in pts with BRAF MT
Laurent-Puig *et al*	169	116 (69%) Res 52/116 (45%)	53 (31%)	5/116 (2.9%) Lower RR (p = 0.63), PFS and OS (p < 0.001)	22/116 (19.8%) Shorter OS (p = 0.013)	na	High EGFR polysomy in 17.7% and correlates with response
Loupakis *et al.*	122 (88 KRAS, 85 PTEN)	53 Res 13/53 (25%)	35/88 (40%) Concordance 95%	na	49/85 (58%) Concordance 60% Higher RR/PFS with KRAS wt/PTEN + mets (p = 0.0004, p = 0.001)	na	pAKT-positive 35/96 (40%) Concordance 68%
Sartore-Bianchi *et al.*	132	43 Res 22/43 (51%)	35 (26.5%)	11 (8.3%) Shorter OS	41 (36%) Lower RR and OS	15 (12.3%) More common in exon 20 Lower RR	KRAS and BRAF mutually exclusive only

Res = responders; na = not assessed; NR = non-responder; Wt = wild type; mt = mutant; RR = response rate; PFS = progression free survival; OS = overall survival; mabs = monoclonal antibodies; Concordance = concordance between primary tumor and metastases.
